# Incidental Transient Cortical Blindness after Lung Resection

**DOI:** 10.1055/s-0036-1571440

**Published:** 2016-02-04

**Authors:** Murat Oncel, Guven Sadi Sunam, Asuman Orhan Varoglu, Hakan Karabagli, Huseyin Yildiran

**Affiliations:** 1 Department of Thoracic Surgery, Selcuk University Medical School, Konya, Turkey; 2 Department of Neurology, Selcuk University Medical School, Konya, Turkey; 3 Department of Neurosurgery, Selcuk University Medical School, Konya, Turkey

**Keywords:** vision loss, cortical blindness, lung resection, diabetes mellitus, cerebral hypoxia

## Abstract

Transient vision loss after major surgical procedures is a rare clinical complication. The most common etiologies are cardiac, spinal, head, and neck surgeries. There has been no report on vision loss after lung resection. A 65-year-old man was admitted to our clinic with lung cancer. Resection was performed using right upper lobectomy with no complications. Cortical blindness developed 12 hours later in the postoperative period. Results from magnetic resonance imaging and diffusion-weighted investigations were normal. The neurologic examination was normal. The blood glucose level was 92 mg/dL and blood gas analysis showed a PO _2_ of 82 mm Hg. After 24 hours, the patient began to see and could count fingers, and his vision was fully restored within 72 hours after this point. Autonomic dysfunction due to impaired microvascular structures in diabetes mellitus may induce posterior circulation dysfunction, even when the hemodynamic state is normal in the perioperative period. The physician must keep in mind that vision loss may occur after lung resection due to autonomic dysfunction, especially in older patients with diabetes mellitus.

Transient visual loss (cortical blindness) in the postoperative period is an uncommon complication and has an incidence varying between 0.05 and 0.1%. 1 Although the exact cause is still speculative, the most likely underlying mechanisms are advanced age, diabetes mellitus, hypertension, smoking, cerebral hypoxia, and procedure duration. 2 This complication is generally associated with cardiac and spine surgeries. 3 Here, we report a patient with diabetes mellitus and bilateral transient visual loss after lung resection. To our knowledge, this case is the first of its kind in the literature.

## Case Report

A 65-year-old man was admitted to our clinic with a mass that was suspected to be lung cancer; it was an undefined mass. At admission, his blood pressure was 120/80 mm Hg. The patient had had type 2 diabetes for ∼12 years, treated intramuscularly with insulin and orally with metformin for 5 years. The patient's medical history showed occasional hypoglycemic attacks and autonomic symptoms. Holding metabolic activity was seen at the right upper lung lobe under positron tomography. Sodium, potassium, phosphorus, blood urine nitrogen, creatine, plasma triglyceride, low-density protein, and high-density lipoprotein were within normal limits. His blood glucose level was 112 mg/dL. A right upper lobectomy was performed for diagnostic purposes. There were no complications perioperatively. Pathology included an abscess and inflammatory process in the mass.

**Fig. 1:**
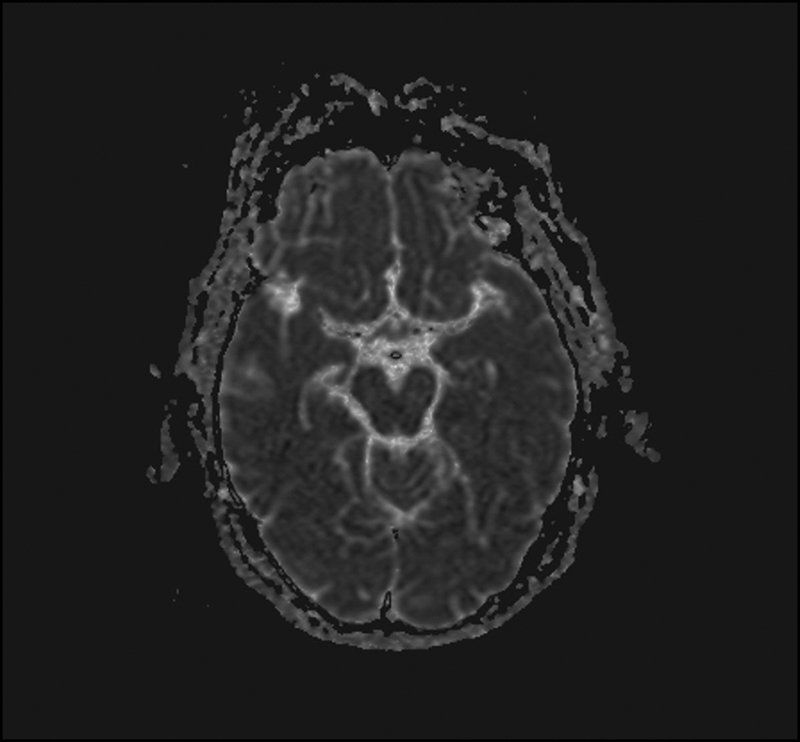
Diffusion-weighted magnetic resonance imaging was normal.

Cortical blindness developed 12 hours postoperatively. The patient's blood glucose level was 92 mg/dL, and blood gas analysis showed a PO _2_ of 82 mm Hg. Cardiac enzymes and electrocardiography were normal. Additionally, the diffusion-weighted magnetic resonance imaging (DWI) was normal ( Fig. 1 ). The patient's neurologic examination was also normal. An ophthalmologic examination showed that the fundus and retina were normal, with no evidence of glaucoma. After 24 hours, the patient could see to count fingers, and full vision was restored within 72 hours of this point.

## Discussion

Cortical blindness is defined as visual loss with normal pupillary reflexes and generally involves the complete return of vision within a few days. 1 Some etiologic factors have been related to cortical blindness in the literature. The most common cause of cortical blindness is cerebral hypoxia. 4 To prevent cerebral hypoxia, it is important to treat severe anemia and avoid hypotension or overtransfusion. 4 Our available records showed no details regarding intraoperative blood loss, hypotension, or anemia.

In the literature, a few cases of cortical blindness after an asthmatic attack have been discussed. In this report, using brain magnetic resonance imaging and single-photon emission computed tomography, signal alterations were demonstrated in the bilateral occipital region. 5 Although there were no signs of respiration stress and the brain DWI was normal, hypoxemia may have contributed to the development of cortical blindness in our case. However, this contribution was not the primary cause of the development of cortical blindness.

Posterior ischemic optic neuropathy is a differential diagnosis of cortical blindness. It is generally seen in ∼60% patients in the first 24 hours postoperatively. 6 In our case, the pupillary reflexes, fundoscopic examination, and brain DWIs were normal. Therefore, we did not think that the visual loss was due to posterior ischemic optic neuropathy.

The other causes of cortical blindness are surgical procedures and prone spine surgery. 7 When the mean operative time exceeds 450 minutes and a patient's position is changed from supine to prone, intraocular pressure increases and visual loss develops. Our patient was operated on using a neutral head position, and the surgery lasted about 2 hours.

The precise pathomechanism of cortical blindness is unknown. Ocular perfusion pressure is the result of the difference between mean blood pressure and intraocular pressure. Hypotension is one of the most frequent causes of cortical blindness. However, hypertension also induces this complication in patients with eclampsia. 8

Transient cortical blindness after coronary angiography is rarely seen. The most likely underlying mechanism is a breakdown of the blood–brain barrier with direct neurotoxicity of the contrast media. 9 The other cause of cortical blindness is posterior reversible encephalopathy syndrome. 10 Therefore, we speculated that circulation and endothelial functions in the occipital region may be vulnerable to blood pressure alteration, which can damage structures other than the brain. In our case, cortical blindness may have resulted from this vulnerability in the occipital region.

Poor glycemic control is believed to play a pivotal role in the pathogenesis of autonomic complication in patients with diabetes mellitus. Its underlying etiology is not well understood. 11 In type 2 diabetes, the autonomic nervous system is impaired due to damage to neurons, sensory receptors, synapses, and blood vessels. Recent studies have shown that autonomic dysfunction is associated with aging. Hypoglycemia is an important sign of autonomic failure and is a life-threatening complication. 12 Severe hypoglycemia is associated with a significant increase in the adjusted risks of major macrovascular and microvascular events. Moreover, minor hypoglycemic attacks can also have serious complications. 12 Our patient had had diabetes mellitus for 12 years and was diagnosed with autonomic neuropathy 2 years previously. He had been treated intramuscularly with insulin and orally with metformin for 5 years, but the patient occasionally described autonomic symptoms in the medical history. The blood glucose level was between 84 and 123 mg/dL, and the blood pressure was between 130/80 and 140/90 mm Hg at the first postoperative day. Cortical blindness developed 12 hours later. Hydration and oxygen were supplied, and the patient was investigated by an endocrinologist. The treatment was initiated immediately according to his suggestions, and the patient then recovered from the cortical blindness. We considered that autonomic complications resulting from diabetes mellitus were the first cause of the cortical blindness in our case.

## Conclusion

Early diagnosis and immediate treatment of cortical blindness are very important because of its potentially permanent effect. We have demonstrated that transient cortical blindness resulted from autonomic complications in a patient with diabetes mellitus. Physicians should be alert to the presence of minor hypoglycemic events and other autonomic complications in diabetes mellitus, particularly in elderly patients who will undergo operation.
